# Supplementary material for: The Design and Immunogenicity of an HIV-1 Clade C Pediatric Envelope Glycoprotein Stabilized by Multiple Platforms
Source: Vaccines (Basel). 2025 Jan 22;13(2):110. doi: 10.3390/vaccines13020110 (PMC11860714; doi:10.3390/vaccines13020110)
Supplement: Supplementary file 1 [file vaccines-13-00110-s001.zip › vaccines-3384305-supplementary.pdf]

	AIIMS_329 SOSIP.v8.2	Comment
501C-605C[a]	[2c6a9a]	Soluble prefusion state stabilization
I559P[a]	[2c6a9a]	
R6 furin site[a]	[2c6a9a]	
ΔMPER[a]	[2c6a9a]	
64K/66R[b]	[2c6a9a]	Decreased exposure of non-nAb V3 epitopes
315Q[b,*]	[2c6a9a]	
316W[b]	[2c6a9a]	
535M[b]	[2c6a9a]	
543N[b]	[2c6a9a]	
73C-561C[c]	[2c6a9a]	Increased thermostability
47D[d]	[2c6a9a]	BG505-derived stabilization
49E[d]	[2c6a9a]	
65K[d]	[2c6a9a]	
165L[d]	[2c6a9a]	
429R[d]	[2c6a9a]	
432Q[d]	[2c6a9a]	
500R[d]	[2c6a9a]	
106E[f,*]	[b46f39]	Increased expression and trimerization
271I[f,*]	[b46f39]	
288L[f,*]	[b46f39]	
304V[f]	[b46f39]	
319Y[f]	[b46f39]	
363Q[f]	[b46f39]	
519S[f]	[b46f39]	
568D[f]	[b46f39]	
570H[f]	[b46f39]	
585H[f]	[b46f39]	
166R[g]	[b46f39]	V2-apex epitope optimization
168K[g]	[b46f39]	
170Q[g]	[b46f39]	
171K[g]	[b46f39]	

Colors	Hexadecimal code
[b46f39]	b46f39
[2c6a9a]	2c6a9a

[a] described in Sanders et al., Plos Pathogens 2013

[b] described in de Taeye et al., Cell 2015. The variant 64K (v4.1) is used for BG505 and B41; while the variant 66R (v4.2) is used

[c] described in Torrents de la Peña et al., Cell Reports 2017. 72C-564C (v5.1) is an alternative disulfide bond that works as well as

[d] described in Guenaga et al., Journal of Virology 2015. All these mutations are naturally present in BG505 wildtype.

[e] described in Guenaga et al., Immunity 2017.

[f] described in Steichen et al., Immunity 2016.

[g] described in Andrabi et al., Immunity 2015.

[*] All these mutations are naturally present in AIIMS_329 wildtype.

Figure S1: Design strategy of 329 SOSIP.v8.2 Env trimer.

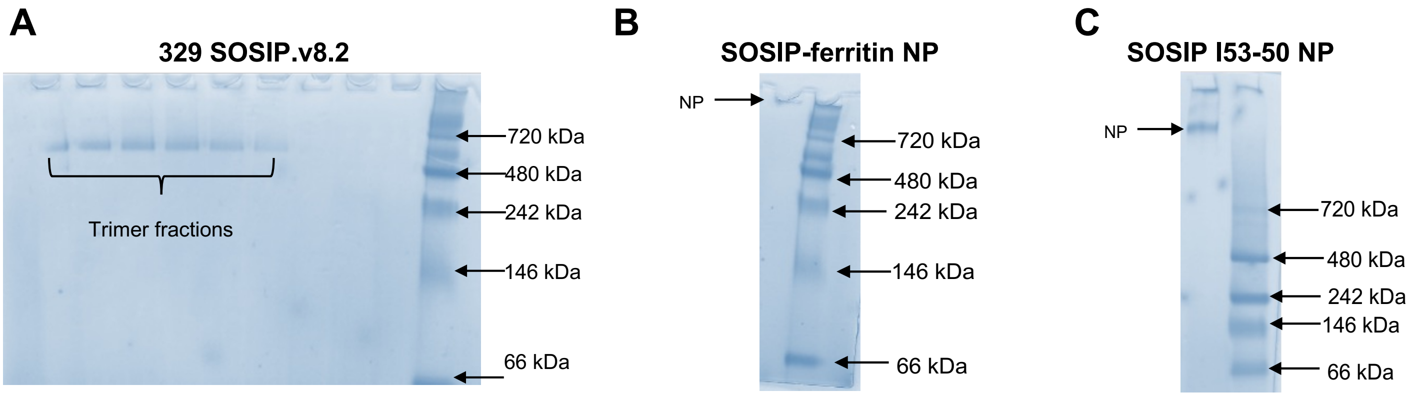

Figure S2: BN-PAGE analysis of 329 Env trimer and assembled NP. A. 2 μg of PGT145 purified 329 SOSIP.v8.2 fractions were analyzed on 4-16% Bis-Tris NativePAGE. **B.** 2 μg of 329 SOSIP-ferritin NP was analyzed on 4-16% Bis-Tris NativePAGE. **C.** 2 μg of assembled 329 SOSIP-I53-50 NP was analyzed on 3-12% Bis-Tris NativePAGE.

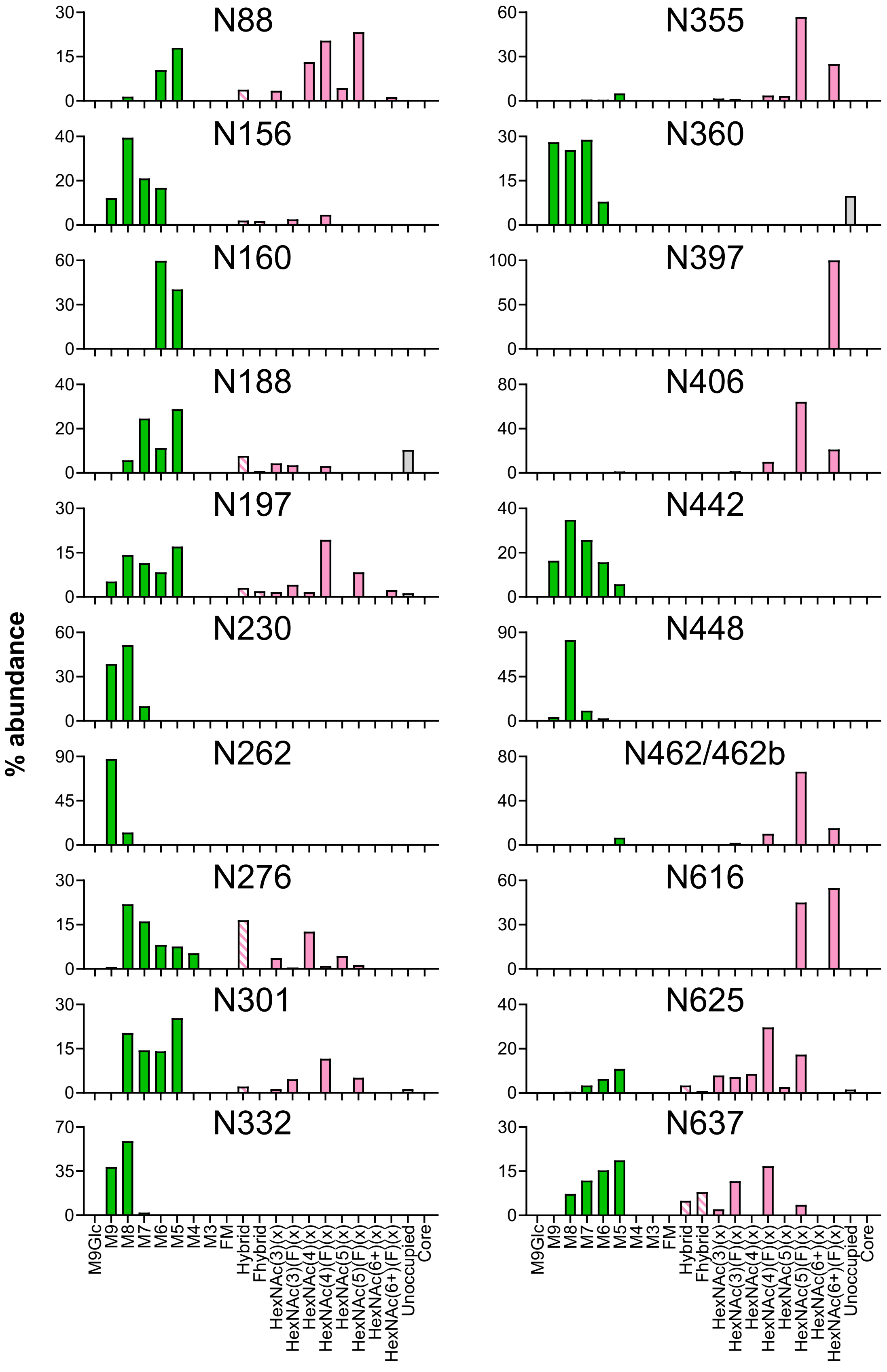

Figure S3: Site-specific glycan analysis of 329 SOSIP.v8.2 Env trimer. Compositional site-specific glycan analysis of 329 SOSIPv8.2 Env trimer. The graphs summarize quantitative mass spectrometric analysis of the glycan population present at individual N-linked glycosylation sites simplified into categories of glycans. The oligomannose-type glycan series (M9 to M5; Man9GlcNAc2 to Man5GlcNAc2) is colored green, afucosylated and fucosylated hybrid-type glycans (hybrid and Fhybrid) are hatched pink, and complex glycans are grouped according to the number of antennae and presence of core fucosylation and are colored pink. Unoccupancy of an N-linked glycan site is represented in grey. The site-specific glycosylation of N462 and N462b could not be resolved but instead the data represent glycans detected at either N462 or N462b.

Table S1: HIV-1 bnAbs, non-nAbs and sCD4 neutralization IC_{50} values (µg/mL) of AIIMS_329 env pseudovirus 329.14.B1.

Epitope regions	HIV-1 mAbs	329.14.B1 virus
V2-apex	PG9	<0.02
	PG16	4.665
	PGDM1400	0.214
	PGT145	>10
	CAP256.25	1.96
V3-glycan	PGT121	<0.004
	BG18	0.078
	PGT128	<0.002
	10-1074	0.008
	AIIMS-P01	0.063
CD4bs	VRC01	0.366
	3BNC117	3.412
	N6	0.063
gp120-gp41 interface	PGT151	2.127
Weakly or non-nAb	2G12	>20
	447-52D	>20
	17b	>20
	48D	>20
	F105	>20
	b6	>20
	19b	>20
	sCD4	>20

Table S2: Site-specific glycosylation of 329 SOSIPv8.2 Env trimers.

AIIMS.329 SOSIPv8.2	N88	N156	N160	N188	N197	N230	N262	N276	N301	N332	N355	N360	N397	N406	N442	N448	N462/N462b	N611	N625	N637
M9Glc	0	0	0	0	0	0	0	0	0	0	0	0	0	0	0	0	0	0	0	0
M9	0	12	0	0	5	39	87	1	0	38	0	28	0	0	16	4	0	0	0	0
M8	1	39	0	6	14	51	13	22	20	59	1	25	0	0	35	82	0	0	1	7
M7	0	21	0	25	11	10	0	16	14	2	1	29	0	0	26	10	0	0	3	12
M6	10	17	60	11	8	0	0	8	14	1	1	8	0	0	16	3	0	0	6	15
M5	18	0	40	29	17	0	0	8	25	0	5	0	0	1	6	1	7	0	11	19
M4	0	0	0	0	0	0	0	5	0	0	0	0	0	0	0	0	0	0	0	0
M3/FM3	0	0	0	0	0	0	0	0	0	0	0	0	0	0	0	0	0	0	0	0
FM	0	0	0	0	0	0	0	0	0	0	0	0	0	0	0	0	0	0	0	0
Hybrid	4	2	0	8	3	0	0	16	2	0	0	0	0	0	0	0	0	0	3	5
Fhybrid	0	2	0	1	2	0	0	0	0	0	0	0	0	0	0	0	0	0	1	8
HexNAc(3)(x)	3	0	0	4	2	0	0	4	1	0	2	0	0	0	0	0	0	0	8	2
HexNAc(3)(F)(x)	0	2	0	3	4	0	0	0	5	0	1	0	0	1	0	0	2	0	7	12
HexNAc(4)(x)	13	0	0	0	2	0	0	13	0	0	0	0	0	0	0	0	0	0	9	0
HexNAc(4)(F)(x)	20	5	0	3	19	0	0	1	12	0	4	0	0	10	0	0	10	0	30	17
HexNAc(5)(x)	4	0	0	0	0	0	0	4	0	0	3	0	0	0	0	0	0	0	3	0
HexNAc(5)(F)(x)	23	0	0	0	8	0	0	1	5	0	57	0	0	64	0	0	66	45	17	4
HexNAc(6+)(x)	0	0	0	0	0	0	0	0	0	0	0	0	0	0	0	0	0	0	0	0
HexNAc(6+)(F)(x)	1	0	0	0	2	0	0	0	0	0	25	0	100	21	0	0	15	55	0	0
Unoccupied	0	0	0	10	1	0	0	0	1	0	0	10	0	0	0	0	0	0	2	0
Core	0	0	0	0	0	0	0	0	0	0	0	0	0	0	0	0	0	0	0	0
Oligomannose	30	89	100	70	56	100	100	60	74	100	8	90	0	2	99	100	7	0	21	53
Hybrid	4	4	0	9	5	0	0	17	2	0	0	0	0	0	0	0	0	0	4	13
Complex	66	7	0	11	37	0	0	24	23	0	92	0	100	98	1	0	93	100	73	34
Unoccupied	0	0	0	10	1	0	0	0	1	0	0	10	0	0	0	0	0	0	2	0
Fucose	45	9	0	7	36	0	0	3	21	0	87	0	100	97	1	0	93	100	55	40
NeuAc	7	0	0	1	6	0	0	7	1	0	18	0	0	27	0	0	10	33	16	2

*Glycans detected for N462 and N462b represent those occupying N462 or N462b

Table S3: Rabbit plasma neutralization ID_{50} titers.
Here N/A refers to 'not applicable' and ND refers to 'not determined'.

				Virus						
				MuLV	RNB1	RTE6	MW965.26	SF162	25710	BG505
				Tier/Clade						
				N/A	ND / C	ND / C	1A	1A	1A	2A
**Study**	**Groups**	**Groups**	**Rabbit ID**	**ID50 titers**						
LBPL/NG-1736 (EF)	Group 1	Placebo	NZW5251	<20	<20	<20	<20	<20	<20	<20
			NZW5252	<20	<20	<20	<20	<20	<20	<20
	Group 2	329 SOSIP	NZW5253	<20	38	100	33	33	33	<20
			NZW5254	<20	58	83	<20	<20	<20	<20
			NZW5255	<20	50	216	27	38	50	<20
			NZW5256	<20	38	122	44	22	38	<20
	Group 3	329 I53-50 NP	NZW5257	<20	61	500	44	38	44	<20
			NZW5258	<20	188	500	133	216	77	<20
			NZW5259	<20	61	88	55	38	27	<20
			NZW5260	<20	150	138	44	33	61	<20
	Group 4	329 ferritin NP	NZW5261	<20	50	122	44	<20	44	<20
			NZW5262	<20	133	200	46	38	55	<20
			NZW5263	<20	82	88	44	33	38	<20
			NZW5264	<20	163	172	38	50	55	<20

ID_{50}
<20
20-100
100-300
300-2000
